# Predictors of long-term neutralizing antibody titers following COVID-19 vaccination by three vaccine types: the BOOST study

**DOI:** 10.1038/s41598-023-33320-x

**Published:** 2023-05-09

**Authors:** Aric A. Prather, Ethan G. Dutcher, James Robinson, Jue Lin, Elizabeth Blackburn, Frederick M. Hecht, Ashley E. Mason, Elena Fromer, Bresh Merino, Remi Frazier, Julia O’Bryan, Stacy Drury, Elissa S. Epel

**Affiliations:** 1 Center for Health and Community, University of California, 675 18th St., San Francisco, CA 94107 USA; 2 Department of Psychiatry and Behavioral Sciences, University of California, San Francisco, USA; 3 Department of Pediatrics, Tulane University School of Medicine, New Orleans, USA; 4 Department of Biochemistry and Biophysics, University of California, San Francisco, USA; 5 Department of Medicine, University of California, San Francisco, USA; 6 Osher Center for Integrative Health, University of California, San Francisco, USA; 7 Academic Research Systems, University of California, San Francisco, USA; 8 Department of Psychiatry, Tulane University School of Medicine, New Orleans, USA

**Keywords:** Vaccines, Human behaviour

## Abstract

As concerns related to the COVID-19 pandemic continue, it is critical to understand the impact of vaccination type on neutralizing antibody response durability as well as to identify individual difference factors related to decline in neutralization. This was a head-to-head comparison study following 498 healthy, community volunteers who received the BNT162b2 (n = 287), mRNA-1273 (n = 149), and Ad26.COV2.S (n = 62). Participants completed questionnaires and underwent blood draws prior to vaccination, 1 month, and 6 months after the vaccination series, and neutralizing antibody (nAB) titers at 1- and 6-months post vaccination were quantified using a high-throughput pseudovirus assay. Over 6 months of follow-up, nABs declined in recipients of BNT162b2 and mRNA-1273, while nABs in recipients of Ad26.COV2.S showed a significant increase. At the 6-month time point, nABs to Ad26.COV2.S were significantly higher than nABs to BNT162b2 and equivalent to mRNA-1273. Irrespective of follow-up timing, being older was associated with lower nAB for participants who received BNT162b2 and Ad26.COV2.S but not for those who received mRNA-1273. A higher baseline BMI was associated with a lower nAB for Ad26.COV2.S recipients but not for recipients of other vaccines. Women and non-smokers showed higher nAB compared to men and current smokers, respectively. The durability of neutralizing antibody responses differed by vaccine type and several sociodemographic factors that predicted response. These findings may inform booster recommendations in the future.

## Introduction

The COVID-19 pandemic, caused by the global spread of the SARS-CoV-2 virus, has led to millions of deaths worldwide. In the United States, the Food and Drug Administration granted emergency use authorization for three vaccines developed against SARS-CoV-2 including the single-dose viral vector vaccine Ad26.COV2.S [Janssen/Johnson & Johnson] and the two-dose mRNA vaccines BNT162b2 [Pfizer/BioNTech] or mRNA-1273 [Moderna]. Randomized controlled trials demonstrated that each of these vaccines provided significant protection against severe disease^1–3^, with neutralizing antibody titers being strongly correlated with protection^4,5^. However, as the pandemic continued, increasing evidence suggests that protection wanes over time^3,6^ and that individual differences in sustained immune protection exist. With the continued persistence of COVID infection waves, there is an urgent need to understand individual-level factors that predict neutralization antibody (nAB) response and durability over time and whether these individual-level factors are predictive of differences in sustained immune protection as a function of vaccine type.

Several studies have identified significant predictors of nAB to COVID-19 vaccination, including evidence of prior infection and chronological age^7–9^. Other factors known to be associated with antibody response are sex, smoking history, and body mass index^10–12^. In this regard, prior studies have shown poorer antibody responses in men^13,14^ and among those who currently smoke^15^. A small study focused on BNT16b2 found that higher BMI was associated with lower antibody titers 6-months post vaccination^10^.

There are limited data comparing the durability of nAB of BNT162b2, mRNA-1273, and Ad26.COV2.S. Prior studies have been relatively small, marked by limited follow up, and rarely examine sociodemographic predictors of nAB response^16–18^. Given the high likelihood for long term reliance on COVID-19 vaccine boosters, data collected in real world settings are needed to inform decisions around which vaccine to deploy and factors that may attenuate vaccine efficacy. To address this gap in the literature, in the present analysis, we report on data from a prospective, observational study that enrolled healthy adults who had not received the COVID-19 vaccine. Baseline sociodemographic and blood measures were obtained prior to vaccination and +1 month and +6 months following vaccination. Here, we provide a head-to-head comparison of BNT162b2, mRNA-1273, and Ad26.COV2.S on nAB over the course of 6-months post-vaccination. We also investigate whether sociodemographic factors predict nAB durability and evaluate the extent to which the impact of these factors varies by vaccine type.

## Results

**Figure 1:**
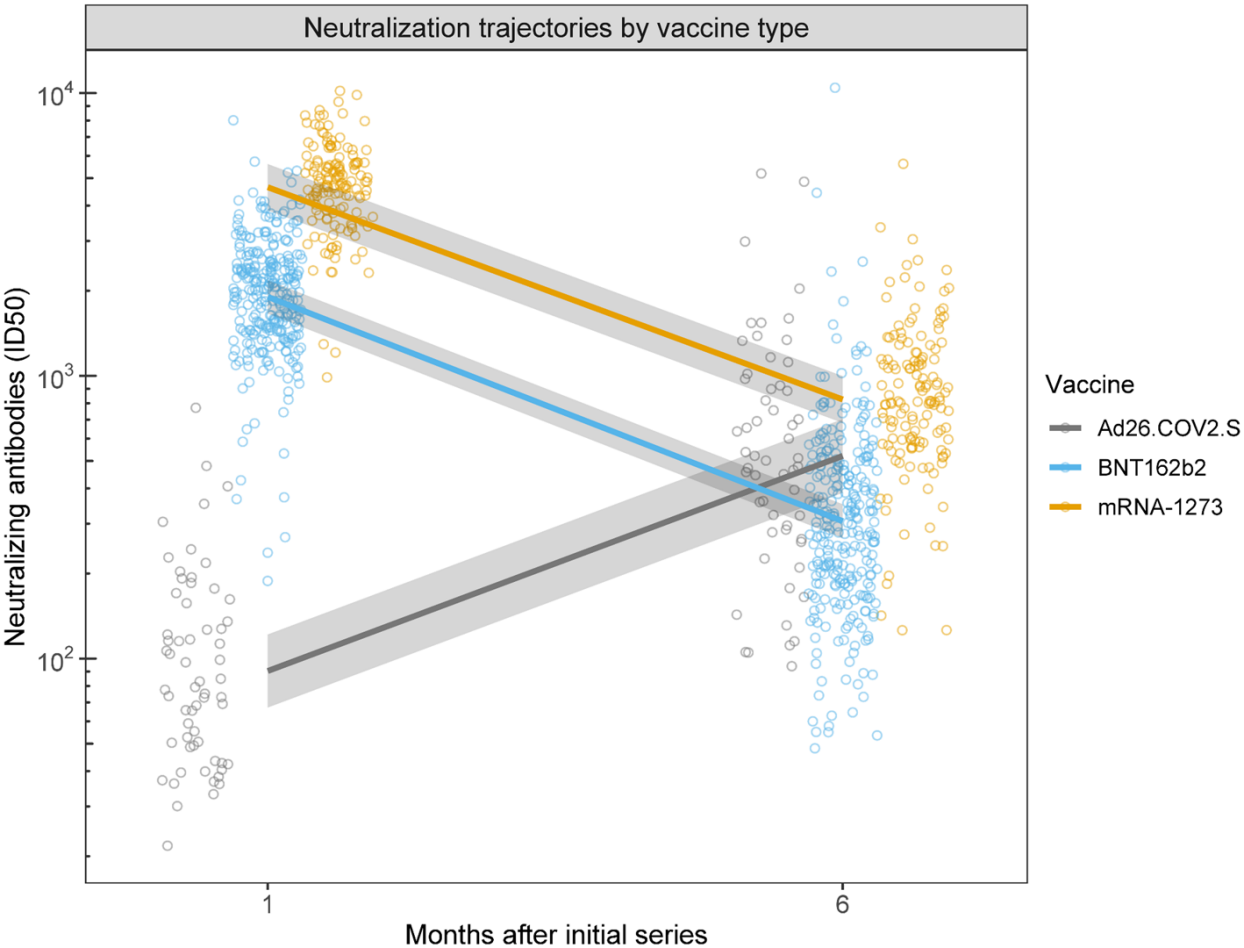
Neutralizing antibodies (nAB) differed by vaccine type. A significant two-way interaction between time and vaccine type was identified. nAB increased over time for Janssen participants while decreasing over time for Moderna and Pfizer participants. At 6 months, Moderna was superior to Janssen, and both Moderna and Janssen were superior to Pfizer. Lines and shading represent model-derived estimated means +/− 95% CI for each combination of time and vaccine.

Descriptive statistics of the study sample are provided in Table 1. Sample characteristics did not differ by vaccine type, except with respect to age. Participants who received Ad26.COV2.S were slightly older as a group compared to those who received the other vaccines. In general, each vaccine led to increased neutralization, measured at the 1 month and 6 month follow-up period. Specifically, at 1 month post-vaccination 94.7% of participants were positive for neutralizing antibody response (nAB) (99.3% BNT162b2, 99.3% mRNA-1273, and 59.7% Ad26.COV2.S). At the 6 month follow-up time point 93.5% were positive (92.2% BNT162b2, 97.9% mRNA-1273, and 89.5% Ad26.COV2.S). Over the course of the 6 month follow up (Fig. 1), nABs declined in recipients of BNT162b2 (0.16-fold; mean difference = − 0.79, CI − 0.85 to − 0.73; *p* < 0.001) and mRNA-1273 (0.18-fold; mean difference = − 0.75, CI − 0.84 to − 0.67; *p* < 0.001), while nABs in recipients of Ad26.COV2.S showed a significant increase (5.8 fold; mean difference = 0.76, CI 0.63 to 0.90; *p* < 0.001).

**Table 1:** Sociodemographic characteristics of the BOOST study sample.

	Full sample (n = 498)	BNT162b2 (n = 287)	mRNA-1273 (n = 149)	Ad26.COV2.2 (n = 62)	*p*-value
Age (years)	55 (46.2–61)	55 (43.5–61)	56 (46–61)	58 (54–62)	0.03
Median, IQR, [range]	[18–88]	[18–76]	[19–72]	[30–88]	
Sex, female, n (%)	320 (64.3%)	188 (65.5%)	94 (63.1%)	38 (61.3%)	0.77
Body mass index (kg/m^2^)	25.7 (23.3–29.9)	25.3 (22.9–28.9)	26.1 (23.4–30.2)	26.2 (24.2–30.2)	0.33
Median, IQR, [range]	[13.5–51.6]	[17–51.5]	[13.5–50.7]	[19.9–51.6]	
Baseline spike IgG (OD)	0.1 (0.1–0.1)	0.1 (0.1–0.1)	0.1 (0.1–0.1)	0.1 (0.1–0.1)	0.56
Median, IQR, [range]	[0–2.6]	[0–2.6]	[0–2.1]	[0.1–2.1]	
Current smoking, n (%)	10 (2.0)	3 (1.0)	4 (2.7)	3 (4.8)	0.12
Race/Ethnicity, n (%)					0.17
Black/African American	17 (3.4)	8 (2.8)	4 (2.7)	5 (8.1)	
Asian	120 (24.1)	76 (26.5)	29 (19.5)	15 (24.2)	
White	277 (55.6)	148 (51.6)	94 (63.1)	35 (56.5)	
Other/Multiracial	32 (6.4)	20 (7.0)	9 (6.0)	3 (4.8)	
Hispanic/Latinx	50 (10.0)	34 (11.8)	12 (8.1)	4 (6.5)	
Educational attainment, n (%)					0.30
Some college or less	77 (15.5)	41 (14.3)	24 (16.1)	12 (19.4)	
4-year degree	197 (39.6)	111 (38.7)	56 (37.6)	30 (48.4)	
Professional degree/Doctorate	224 (45.0)	135 (47.0)	69 (46.3)	20 (32.3)	
Household income, n (%)					0.75
Less than $50,000	61 (12.2)	30 (10.5)	22 (14.8)	9 (14.5)	
$50,000 to less than $100,000	96 (19.3)	61 (21.3)	25 (16.8)	10 (16.1)	
$100,000 to less than $200,000	150 (30.1)	87 (30.3)	41 (27.5)	22 (35.5)	
$200,000 or more	125 (25.1)	73 (25.4)	38 (25.5)	14 (22.6)	
Prefer not to answer	66 (13.3)	36 (12.5)	23 (15.4)	7 (11.3)	
Marital status, n (%)					0.06
Married or with a long-term partner	288 (57.8)	160 (55.7)	89 (59.7)	39 (62.9)	
Never married	148 (29.7)	95 (33.1)	42 (28.2)	11 (17.7)	
Divorced or separated	51 (10.2)	26 (9.1)	16 (10.7)	9 (14.5)	
Widowed	11 (2.2)	6 (2.1)	2 (1.3)	3 (4.8)	

*IQR* Interquartile range.

We compared the neutralization antibody responses by vaccine type at each follow-up time point. At the 1-month follow-up, nABs to BNT162b2 and mRNA-1273 were 51-fold (*p* < 0.001) and 21-fold (*p* < 0.001) higher than those generated in response to Ad26.COV2.S. nABs were 2.4-fold higher in recipients of mRNA-1273 compared to those who received BNT162b2 (*p* < 0.001). At the 6-month follow-up, Ad26.COV2.S recipients showed 1.7-fold higher nAB compared to BNT162b2 recipients (*p* < 0.001) and 0.63-fold lower nAB compared to mRNA-1273 recipients (*p* = 0.01). nABs were 2.7-fold higher in those who received mRNA-1273 compared to BNT162b2 at the 6-month follow-up (*p* < 0.01). Sensitivity analyses restricted to participants who showed no evidence of prior infection at baseline or during the study (n = 464; 93.2% of the sample) produced similar findings (Table S3).

**Figure 2:**
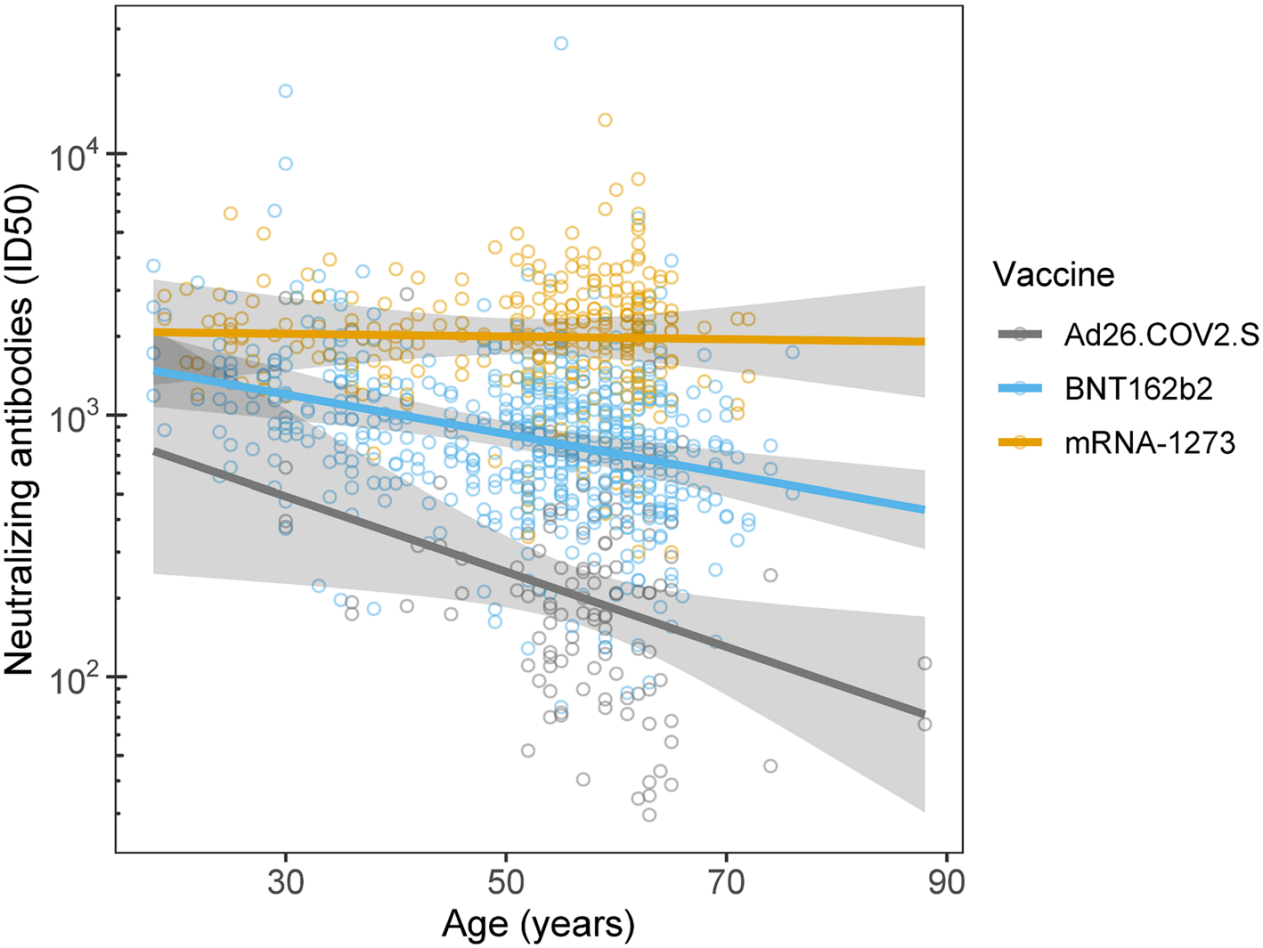
Age interacted with vaccine to predict neutralizing antibody level (nAB). The relationship between age and nAB was most negative for Janssen recipients, then Pfizer recipients, and not significant for Moderna recipients. This hierarchy of vulnerability to age mirrored the hierarchy of nAB produced at 1-month follow-up irrespective of age (Fig. 1), suggesting that vaccine potency may have conferred protection against the effects of age. Lines and shading represent model-derived estimated means +/− 95% CI for all observed levels of age. These estimates represent the effect of age within vaccine irrespective of follow-up time point, because the three-way interaction between age, vaccine, and time point was not significant.

**Figure 3:**
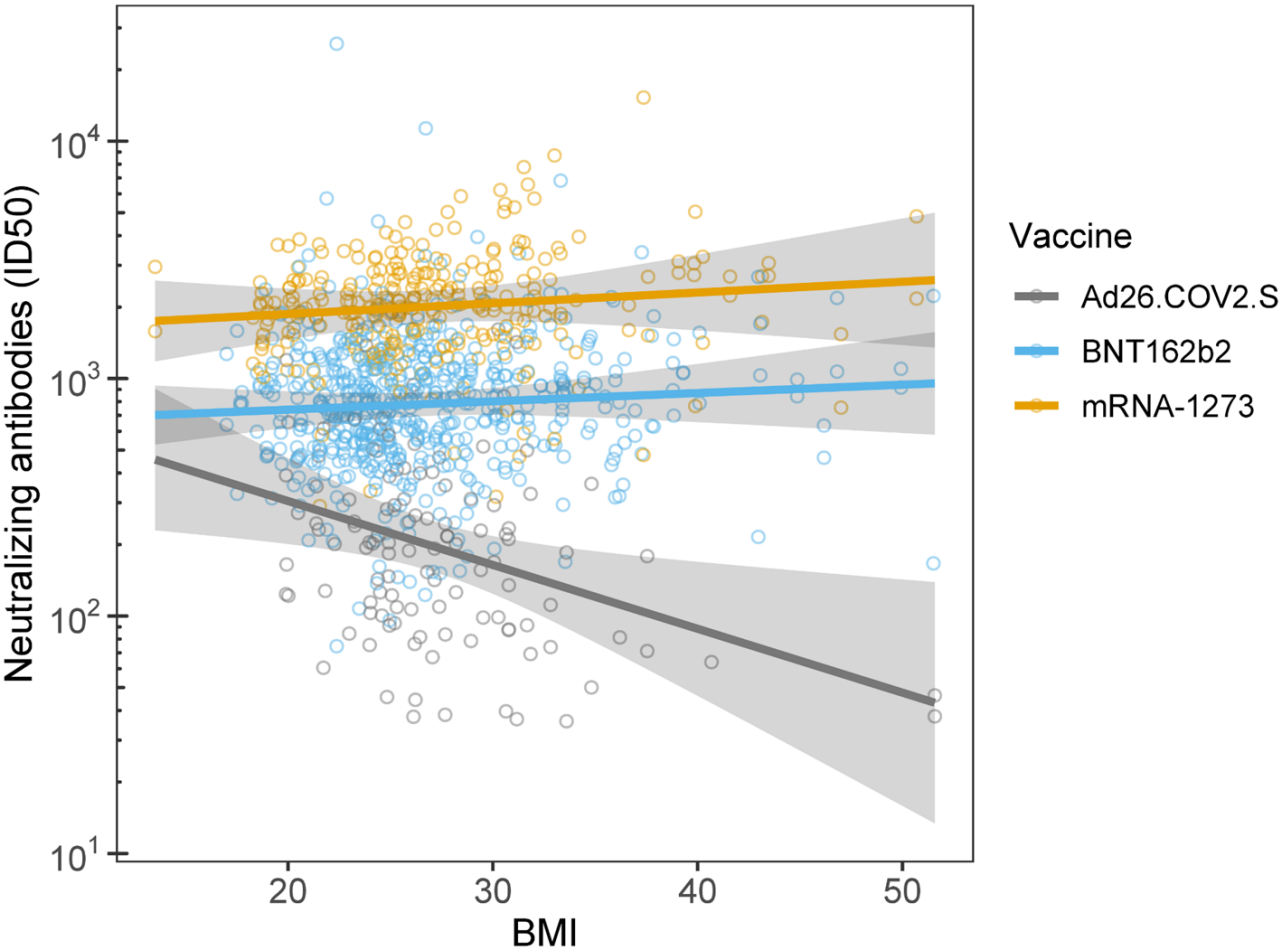
Body mass index (BMI) interacted with vaccine to predict neutralizing antibody level (nAB). BMI exhibited a significant relationship with nAB for Janssen participants but not for either of the mRNA vaccines. Lines and shading represent model-derived estimated means +/− 95% CI for all observed levels of BMI. These estimates represent the effect of BMI within vaccine irrespective of follow-up time point, because the three-way interaction between BMI, vaccine, and time point was not significant.

**Figure 4:**
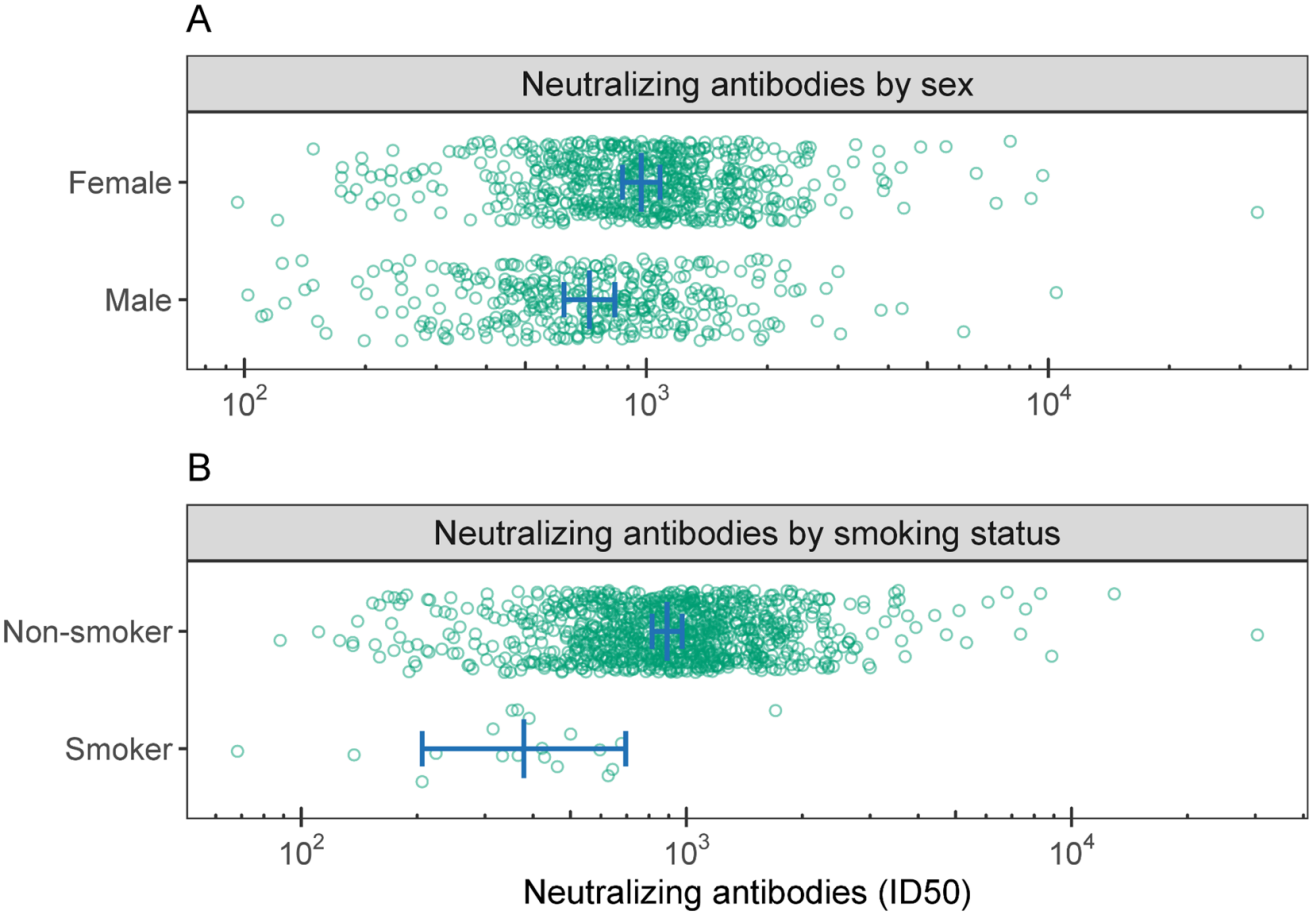
(**A**) and (**B**) Sex and smoking status both predicted neutralizing antibody level (nAB). Higher nAB were observed in females compared to males (upper panel), and non-smokers compared to smokers (lower panel). Lines and error bars represent model-derived estimated means +/− 95% CI. These estimates represent effects irrespective of time point, because no interactions involving sex or BMI were significant.

Several individual-level factors predicted the durability of nAB over the course of the 6-month follow-up. Indeed, being older was associated with overall lower nAB for participants who received the BNT162b2 (r_p_ = − 0.17, CI − 0.26 to − 0.08; slope = − 0.0076, CI − 0.011 to − 0.0037; *p* < 0.001) and Ad26.COV2.S (r_p_ = − 0.11, CI − 0.19 to − 0.02; slope = − 0.014, CI − 0.026 to − 0.0027; *p* = 0.02), but not for mRNA-1273 recipients (r_p_ = − 0.01, CI − 0.10 to 0.08; slope = − 0.00052, CI − 0.0061 to 0.0051; *p* = 0.86; Fig. 2). This interaction between age and vaccine type in predicting nABs was independent of follow-up time point. Similarly, independent of follow-up time point, higher baseline BMI was associated with overall lower nAB for Ad26.COV2.S (r_p_ = − 0.11, CI − 0.20 to − 0.03; slope = − 0.027, CI − 0.047 to − 0.0065; *p* = 0.010), but not mRNA-1273 (r_p_ = 0.04, CI − 0.05 to 0.12; slope = 0.0045, CI − 0.0068 to 0.016; *p* = 0.43) or BNT162b2 (r_p_ = 0.04, CI − 0.05 to 0.13; slope = 0.0035, CI − 0.0050 to 0.012; *p* = 0.42; Fig. 3). There was a main effect of sex, such that women had 1.3-fold higher nAB than men (mean difference = 0.13, CI 0.052 to 0.21; *p* < 0.001; Fig. 4A), irrespective of vaccine type or timepoint. Similarly, non-smokers had 2.4-fold higher nAB compared to smokers (mean difference = − 0.37, CI − 0.64 to − 0.10; *p* = 0.007; Fig. 4B). Finally, baseline anti-spike antibody level, a proxy for prior infection, was associated with higher nAB post-vaccination for each combination of vaccine and time point except with the exception of the 6-month follow-up time point in Ad26.COV2.S recipients (Figure S1).

## Discussion

Vaccines continue to be the primary mitigation strategy for protecting populations against COVID-19. Data on comparability of vaccine immunogenicity are limited; however, our findings are largely consistent with prior serological investigations^16,18–20^. This study found that while mRNA vaccines (mRNA-1273, BNT162b2) produced larger nAB responses initially compared to the adenovirus vector-based vaccine Ad26.COV2.S, nABs waned significantly over 6 months. In contrast, those who received Ad26.COV2.S showed an increase in neutralization over the same period such that at the 6 month follow-up time point Ad26.COV2.S recipients showed significantly higher nABs compared to those who received BNT162b2. Recipients of mRNA-1273 also showed nABs higher than BNT162b2 recipients and were not significantly different from Ad26.COV2.S. Prior studies support the maintenance of nABs to the single dose Ad26.COV2.S at least out to 8 months post-vaccination^16,18,20–22^. However, only one other study has reported an increase in nABs over 6 months post-vaccination in Ad26.COV2.S recipients^18^. Notably, in that study, despite the increase over time, nABs to Ad26.COV2.S remained lower than those generated by mRNA vaccines. With regards to differences between mRNA-1273 and BNT162b2, and consistent with the present study findings, several reports indicate that mRNA-1273 results in more robust protection against future infection^23,24^, and higher nABs at least 6 months post-vaccination^16,25,26^. Together, our findings suggest that while mRNA vaccines generate a robust initial nAB response, mRNA-1273 nABs are maintained at high levels despite waning over time and neutralizing responses to Ad26.COV2.S appear to largely catch up by 6 months post-vaccination in a community sample free from immune system conditions. This study also revealed several individual-level factors that were associated with nAB durability across vaccine types. Indeed, we found that being male and being a smoker were associated with lower nABs irrespective of vaccine type. While the smoking rates in this sample were low (2%), current tobacco use was associated with impaired neutralization, which is consistent with findings from other studies that have focused on antibody responses to various COVID-19 vaccines^12,15,27,28^ and findings in other vaccines more generally^29,30^. There is growing evidence for sex differences in immune responses, including adaptive immunity to SARS-CoV-2. Prior work supports higher antibody titers 1 and 3 months post COVID-19 vaccination in women compared to men^31,32^. At odds with these findings is a recent meta-analysis of pooled clinical trial data that suggests a slight advantage for men when it comes to vaccine efficacy^33^. Future studies are needed that are powered to test sex differences and include different reproductive stages (e.g., during pregnancy, perimenopause, post-menopause).

Other individual differences, including age and BMI, exerted effects that differed by vaccine type and were not specific to a particular follow-up time point in this study. Older age was a strong predictor of nABs over the course of the study, though this was not a relevant factor for mRNA-1273 recipients. This is perhaps not surprising given the large amount of data supporting the robustness of mRNA-1273 in the population^1,34,35^. Further, observational data suggest that mRNA-1273 recipients were less susceptible to breakthrough infections and hospitalizations during the Delta variant surge compared to those who received BNT162b2, which may be clinically relevant given that more severe outcomes track with increasing age. Notably, our neutralization assay was specific to the Wuhan strain, where the effects of age on nABs appear more pronounced compared nABs to other variants (e.g., P.1 variant)^36^.

Our analyses also revealed a negative relationship between baseline BMI and nABs among Ad26.COV2.S recipients. Higher BMI is a risk factor for negative COVID-19 outcomes, including elevated risks for hospital admission and death^37,38^. Further, a study of healthcare workers found that higher BMI was associated with lower spike antibodies to BNT162b2 collected 2 months post-vaccination in men but not women^11^. To our knowledge, this study is the first to compare the influence of BMI on nABs by vaccine type. And while our study was not powered to test the interaction, exploratory analyses suggest that among Ad26.COV2.S recipients, the influence of BMI on nABs was similar between men and women in this sample (data not shown).

Comparability of available vaccines is important for informing vaccine decisions as populations contend with an endemic COVID-19. The present study provides preliminary evidence for surprising differences by vaccines when examining 6-month durability; however, this study is not without limitations. First, this is an observational study and thus participants were not randomized to vaccine type, and while we accounted for key confounders in our study design and statistical analyses, unmeasured confounding is possible. Further, because the study enrolled participants who were free to choose their vaccine, this resulted in a much smaller group who received the Ad26.COV2.S vaccine. Second, we did not investigate neutralizing antibody titers to any SARS-CoV-2 variants. Current vaccine formulations appear less protective against emerging variants, producing more variability in nAB over time^19,39–41^. Third, we do not have data points beyond 6 months, and as such do not know whether differences in vaccines will persist. Fourth, this study focused solely on humoral immunity. Prior work implicates cellular immunity as critical to sustained protection against COVID-19^42^, and early evidence suggests that vaccines may differentially support the durability of cell-mediated immunity, including memory B cells^18^. Relatedly, our analyses were limited to sociodemographic predictors and did not include biological factors that could further enhance nAB prediction. Indeed, recent data support innate transcriptional pathways, including those responsible for regulating inflammation and cell proliferation, that may reflect pre-vaccination immune signatures relevant to vaccine-induced antibody response^43,44^. The incorporation of biological data will be important in building a more robust predictive model of nAB durability. Fifth, a small percentage of participants (< 10%) either declined to provide access to their vaccine card for verification or turned in their vaccine card prior to the 6-month follow-up time point. This limits our ability to confirm that these participants did not receive vaccine boosters prior to the 6-month time point. That said, sensitivity analyses excluding these participants produce results that are in line with our overall findings (Table S4). Finally, it is unclear whether these findings will provide insights into nABs to subsequent vaccine boosters. Given the high likelihood that COVID boosters will be recommended into the future, research characterizing nAB trajectories over time is warranted.

In sum, across the three vaccines compared in this study, mRNA vaccines produced more robust initial responses that waned over time while the adenovirus vector-based vaccine increased over the course of the study. We also identified several individual risk factors associated with the durability of response (sex, smoking, age, and BMI). As more studies are carried out, comparing adenovirus vector-based vaccines and mRNA-based vaccines, these findings may help inform when and for whom vaccine boosters are indicated.

## Methods

The Building Optimal antibOdies STudy (BOOST) study recruited 534 healthy adults who had not yet received a COVID-19 vaccine to participate in an observational study designed to identify psychological, behavioral, and biological predictors of immune response to the COVID-19 vaccination series. Participants were recruited from throughout the San Francisco Bay Area from March 6 to April 17, 2021. Eligible individuals were 18 years or older, had not yet received a COVID-19 vaccine, and were willing to complete questionnaires and blood draws at baseline prior to vaccination, and 1 month (30 days, interquartile range 28–32 days) and 6 months (180 days, interquartile range 178–182 days) following final vaccine injection. Exclusionary criteria included current pregnancy, history of immune-related disease, including autoimmune conditions, viral hepatitis, HIV, and current cancer treatment. Individuals were also excluded if they were taking medications known to affect the immune system, including immunomodulators and corticosteroids. Participants with a prior history of SARS-CoV-2 infection were not excluded. Sensitivity analyses excluding individuals with evidence of infection prior to vaccination or in the vaccination follow-up window can be found Table S3. Study participants independently arranged to be vaccinated and then forwarded information regarding their vaccination to the research team. Study participants were compensated $100 for each of the three assessments. The study was approved by the Institutional Review Board at the University of California, San Francisco, and written, informed consent was obtained from each study participant. We followed the Strengthening the Reporting of Observational Studies in Epidemiology (STROBE) reporting guidelines. All methods were performed in accordance with the relevant guidelines and regulations by a statement provided by Institutional Review Board at the University of California, San Francisco. Sociodemographic and behavioral factors of interest, including age, sex, and smoking status (current tobacco use, yes/no) were obtained by self-report. Body height and weight were measured at the time of the baseline blood draw for body mass index (BMI) calculation. Study data were collected and managed using REDCap electronic data capture tools hosted at the University of California, San Francisco^45,46^.

### Neutralization and SARS-CoV-2 spike/nucleocapsid protein assays

SARS-CoV-2 neutralizing antibody responses (nAB) were assessed in serum with sensitive, high-throughput pseudovirus assays. Pseudoviruses were produced by co-transfection of 293 T cells with a plasmid expressing full-length spike protein of the Wuhan-1 strain containing the D614G amino acid chain (VRC7480.G614)^47^, a pCMV ΔR8.2 lentivirus backbone plasmid (VRC5602)^48^ the VRC5601 plasmid pHR' CMV Luc containing the firefly luciferase reporter gene17, and VRC9260 for TMPRSS2 expression. Virus stocks are collected 3 days after transfection, clarified, passed through a 0.45 μm filter, and stored in aliquots at − 80 °C. For neutralization, a predetermined optimal dose of pseudovirus was incubated with serial threefold dilutions of heat-inactivated serum in 150 μl medium for 1 h at 37 °C in 96-well tissue culture plates. CHO/ACE2 cells, suspended by the action of TrypLE enzyme, were added to wells (10,000 cells in 100 μL medium per well), with appropriate controls. After 66–72 h of incubation, the medium was removed, and 100 ul of 1:6 dilution of Promega BriteGlo in Glo lysis buffer was added. Plates were incubated for 7 min at room temperature, after which luminescence was measured in a Biotek Synergy H1 Luminometer. Neutralizing antibody titers were expressed as the inhibitory dose 50 (ID50), defined as the serum dilution at which relative luminescence units (RLU) would be reduced by 50% compared to virus control wells after subtraction of background RLUs.

Antibodies to the SARS-CoV-2 spike protein at baseline were quantified by ELISA. Briefly, 96-well ELISA plates (Costar, Easy Wash) were coated for 1 h at room temperature with purified SARS CoV-2 spike protein (500 ng/ml in 100 mM sodium bicarbonate buffer). Wells were washed and blocked for 1 h at + 37 °C. Blocking and dilution buffers consisted of 0.5% Tween, 5% dry milk, 4% whey protein (BiPro, Le Sueur, MN), and 10% FBS in 1xPBS. After wells had been washed and blocked, 100 ul heat-inactivated sera samples diluted to 1:100 were incubated in antigen-coated and uncoated wells for 1 h. The wash buffer consisted of PBS with 500 mM NaCl and 0.2% Triton X. The higher salt content of the wash buffer greatly reduced background noise. Bound IgG was detected with peroxidase-conjugated goat anti-IgG (Jackson ImmunoResearch) and diluted at 1:15,000. The color was developed with TMB-H202 and stopped with 1 M phosphoric acid. Absorbance (optical density [OD]) was read at 450 nm. OD values > 0.5 were considered positive. The spike protein ELISA for IgG antibodies has been validated by testing a standard set of positive and negative samples provided by NCI SeroNet staff. These validations showed sensitivity and specificity for the immunoassay as 98% and 100%, respectively. Antibodies to the nucleocapsid (N) protein was quantified at the 1- and 6-month time points using a commercial N protein ELISA according to the manufactures protocol (Zalgen Labs, Germantown, MD) OD values of > 0.8 were considered positive.

### Data analysis

All data processing, graphing, and statistical analysis were performed using R v4.1.3^49^. A base 10 log transformation was applied to the neutralization data, and then a linear mixed-effects model was fit. Of the 534 individuals who presented for blood collection at baseline, 498 individuals contributed to the fit of the regression models, given that they also reported for at least one follow-up time point (providing 496 observations of nAb at the 1-month time point, and 480 observations at the 6-month time point) and provided all necessary predictor data. Missingness reasons for 1-month follow-up were: unknown BMI (n = 19), no-show (n = 12), unknown vaccine (n = 5), unknown baseline smoking status (n = 1), unknown baseline anti-spike IgG (n = 1). Missingness reasons for the 6-month follow-up were: no-show (n = 22), unknown BMI (n = 18), received a booster shot beforehand (n = 6), sample loss (n = 6), unknown baseline smoking status (n = 1), unknown baseline anti-spike IgG (n = 1). There were no significant differences in the composition of the baseline (n = 534) and analytic sample (n = 498) apart from educational attainment, with a slightly higher percentage in the baseline sample reporting an education of “some college or less” than in the analytic sample (Table S1). Given the lack of available data at the onset of this study, no a priori power analysis was conducted.

The initial model allowed the effect of each vaccine to vary over time depending on five predictors: age, gender, BMI, smoking status, and baseline anti-spike IgG. It, therefore, consisted of three-way interactions between the vaccine, timepoint, and five predictors, as well as all lower-order two-way interactions and main effects. Multicollinearity was identified in this fitted model and was resolved by removing the two interactions involving smoking status, resulting in the final model (see Table S2 for an enumeration of all final model terms). The final model met assumptions of residual normality, linearity, and equality of variance. No single observation had an undue influence on model fit, given that all had a Cook’s distance below 0.5. Given the inclusion of a three-level categorical predictor (vaccine), predictor significance was tested using F statistics. F statistics used Type II sums of squares, meaning they compared the model predictions using a specific predictor but without any higher-order predictors to the model predictions without the specified predictor^50^. Visualizations represent the model-derived estimated mean (+/− 95% confidence interval, CI) of nAB for all observed levels of the depicted predictors. Post-hoc testing made the same assumptions Standardized effect sizes, i.e., partial eta squared and partial r, and absolute effect sizes were provided. Detailed information on statistical analysis can be found in the Supplementary Information.

## Supplementary Information

Supplementary Information.

## Data Availability

The data are available from corresponding authors upon reasonable request.
