# Supplementary material for: Predictors of long-term neutralizing antibody titers following COVID-19 vaccination by three vaccine types: the BOOST study
Source: Sci Rep. 2023 May 9;13:6505. doi: 10.1038/s41598-023-33320-x (PMC10170073; doi:10.1038/s41598-023-33320-x)
Supplement: Supplementary file 1 — Supplementary Information. [file 41598_2023_33320_MOESM1_ESM.docx]

**Supplementary Methods**

Data analysis

A linear mixed-effects model was fit to log10 transformed ID50 values using *lme4* v1.1-30, using restricted maximum likelihood estimation. Categorical predictors (vaccine, gender, smoking status, time) were included via deviation coding, and continuous predictors were mean centered prior to model fitting. Random effects were limited to a random intercept per subject. Variance inflation factors were calculated using *car* v3.1-0 and revealed multicollinearity between all interaction terms involving smoking status and the corresponding lower-order terms not including smoking status, which resulted from the very small number of smokers relative to non-smokers. Therefore, interaction terms involving smoking were removed from the model, resolving all multicollinearity. Additional assumptions of residual normality, linearity, and equality of variance were checked using diagnostic plots and were all met.

Predictor significance was tested using Type II sums of squares F-statistics calculated using *car*, with degrees of freedom approximated via the Kenward-Roger method. For visualizations and post-hoc testing, *emmeans* v1.7.5 was used to calculate either model-estimated marginal means or marginal slopes, for each level of a given categorical predictor or combination of two categorical predictors. The levels of any other categorical predictors were weighted proportionally to their representation in the sample in calculation of these marginal means or slopes. For continuous predictors, all estimates assumed the median value. For hypothesis testing, these values were then either compared to one another using a two-sample t-test, or to zero using a one-sample t-test. The *effectsize* package v0.7.0 was used to convert t or Type II sums or squares F statistics and associated degrees of freedom to standardized effect sizes, specifically partial r or partial η^2^.

All visualizations were produced using *ggplot2* v3.3.6 and *patchwork* v1.1.1 and represent marginal means +/- 95% confidence intervals (CIs). Partial residuals are also depicted, which were calculated using *ggeffects* v1.1.2.

Comparisons of demographic characteristics according to vaccine type or missing data status were performed by use of Kruskal-Wallis tests for continuous variables and chi-squared tests for categorical variables.

|  | **All subjects** | **Included in models** | **p value** |
| --- | --- | --- | --- |
| Total n | 534 | 498 |  |
| Age (years)  Median (IQR) [range] | 55 (47-61)  [18-89] | 55 (46.2-61)  [18-88] | 0.81 |
| Sex, Female, n (%) | 343 (64.2) | 320 (64.3) | 1 |
| Body mass index (kg/m2)  Median (IQR) [range] | 25.7 (23.2-29.8)  [13.5-51.6] | 25.7 (23.3-29.9)  [13.5-51.6] | 0.79 |
| Baseline spike IgG (OD),  Median (IQR) [range] | - 1. (0.1-0.1)   [0-2.9] | 0.1 (0.1-0.1)  [0-2.6] | 0.9 |
| Current smoking, n (%) | 10 (1.9) | 10 (2.0) | 0.87 |
| Race/ethnicity, n (%) |  |  | 0.36 |
| Black/African American | 19 (3.6) | 17 (3.4) |  |
| Asian | 129 (24.2) | 120 (24.1) |  |
| White | 294 (55.1) | 277 (55.6) |  |
| Other/Multiracial | 37 (6.9) | 32 (6.4) |  |
| Hispanic/Latinx | 52 (9.7) | 50 (10.0) |  |
| Educational attainment, n (%) |  |  | 0.05 |
| Some college or less | 87 (16.3) | 77 (15.5) |  |
| 4-year degree | 205 (38.4) | 197 (39.6) |  |
| Professional degree or doctorate | 241 (45.1) | 224 (45.0) |  |
| Household income, n (%) |  |  | 0.75 |
| Less than $50,000 | 66 (12.4) | 61 (12.2) |  |
| $50,000 to less than $100,000 | 105 (19.7) | 96 (19.3) |  |
| $100,000 to less than $200,000 | 159 (29.8) | 150 (30.1) |  |
| $200,000 or more | 131 (24.5) | 125 (25.1) |  |
| Prefer not to answer | 71 (13.3) | 66 (13.3) |  |
| Relationship status, n (%) |  |  | 0.47 |
| Married or with a long-term partner | 309 (57.9) | 288 (57.8) |  |
| Never married | 159 (29.8) | 148 (29.7) |  |
| Divorced or separated | 52 (9.7) | 51 (10.2) |  |
| Widowed | 13 (2.4) | 11 (2.2) |  |
| Vaccine, n (%) |  |  | 0.13 |
| Pfizer | 303 (56.7) | 287 (57.6) |  |
| Moderna | 151 (28.3) | 149 (29.9) |  |
| Janssen | 65 (12.2) | 62 (12.4) |  |

**Table S1. Sociodemographic variables for the BOOST sample comparing the entire sample with baseline measures (n=534) with those included in the analytic sample (n=498).**

| **Predictor** | **Partial η_p_^2^** | **F** | **Df** | **p** | **Signif** |
| --- | --- | --- | --- | --- | --- |
| Age | 0.03 (0.01 to 0.06) | 14.23 | 1,482.4 | 2e-04 | *** |
| Baseline S IgG | 0.14 (0.09 to 0.2) | 83.08 | 1,502.5 | 2e-18 | *** |
| BMI | 0 (0 to 0.01) | 0.06 | 1,480.1 | 0.81 |  |
| Sex | 0.02 (0 to 0.06) | 11.11 | 1,481.3 | 9e-04 | *** |
| Smoking status | 0.01 (0 to 0.04) | 7.46 | 1,506.2 | 0.007 | ** |
| Timepoint | 0.62 (0.57 to 0.66) | 766.19 | 1,471.3 | 8e-101 | *** |
| Timepoint × Age | 0 (0 to 0) | 0.00 | 1,473.5 | 1.00 |  |
| Timepoint × Baseline S IgG | 0.01 (0 to 0.03) | 2.56 | 1,485.6 | 0.11 |  |
| Timepoint × BMI | 0.01 (0 to 0.03) | 4.36 | 1,471.2 | 0.037 | * |
| Timepoint × Sex | 0 (0 to 0.02) | 1.01 | 1,472.5 | 0.31 |  |
| Vaccine | 0.36 (0.29 to 0.42) | 135.87 | 2,486.5 | 1e-47 | *** |
| Vaccine × Age | 0.01 (0 to 0.04) | 3.22 | 2,492.2 | 0.041 | * |
| Vaccine × Baseline S IgG | 0.01 (0 to 0.02) | 1.76 | 2,551.2 | 0.17 |  |
| Vaccine × BMI | 0.02 (0 to 0.04) | 3.95 | 2,490.4 | 0.020 | * |
| Vaccine × Sex | 0.01 (0 to 0.03) | 1.68 | 2,489.4 | 0.19 |  |
| Vaccine × Timepoint | 0.5 (0.44 to 0.55) | 240.16 | 2,476.5 | 7e-73 | *** |
| Vaccine × Timepoint × Age | 0 (0 to 0.02) | 0.69 | 2,484.8 | 0.50 |  |
| Vaccine × Timepoint × Baseline S IgG | 0.02 (0 to 0.05) | 6.08 | 2,534.2 | 0.002 | ** |
| Vaccine × Timepoint × BMI | 0.01 (0 to 0.02) | 1.31 | 2,482.6 | 0.27 |  |
| Vaccine × Timepoint × Sex | 0 (0 to 0) | 0.05 | 2,481.4 | 0.95 |  |

**Table S2**. **Model structure**. The terms included in the mixed-effects ANCOVA are provided. Partial eta squared (η_p_^2^) statistics were calculated F statistics and corresponding degrees of freedom (Df). F statistics were calculated using Type II sums of squares, and thus each compares the model with a specific predictor but without any higher-order predictors to the model without

| **Predictor** | **Partial η_p_^2^** | **F** | **Df** | **p** | **Signif** |
| --- | --- | --- | --- | --- | --- |
| Age | 0.03 (0.01 to 0.07) | 15.20 | 1,435.1 | 1e-04 | *** |
| Baseline S IgG | 0 (0 to 0.01) | 0.22 | 1,441.1 | 0.64 |  |
| BMI | 0 (0 to 0.01) | 0.11 | 1,434.8 | 0.75 |  |
| Sex | 0.03 (0 to 0.06) | 11.80 | 1,434.9 | 7e-04 | *** |
| Smoking status | 0.02 (0 to 0.06) | 10.81 | 1,452.8 | 0.001 | ** |
| Timepoint | 0.65 (0.61 to 0.69) | 809.75 | 1,431.7 | 5e-101 | *** |
| Timepoint × Age | 0 (0 to 0.01) | 0.15 | 1,431.6 | 0.70 |  |
| Timepoint × Baseline S IgG | 0 (0 to 0.01) | 0.14 | 1,437.4 | 0.71 |  |
| Timepoint × BMI | 0.01 (0 to 0.04) | 4.13 | 1,431.2 | 0.043 | * |
| Timepoint × Sex | 0 (0 to 0.01) | 0.32 | 1,431.5 | 0.57 |  |
| Vaccine | 0.38 (0.31 to 0.44) | 135.21 | 2,437.8 | 2e-46 | *** |
| Vaccine × Age | 0.02 (0 to 0.05) | 4.34 | 2,439.5 | 0.014 | * |
| Vaccine × Baseline S IgG | 0 (0 to 0.02) | 0.59 | 2,439.7 | 0.55 |  |
| Vaccine × BMI | 0.02 (0 to 0.05) | 4.68 | 2,437.8 | 0.010 | ** |
| Vaccine × Sex | 0 (0 to 0.02) | 0.69 | 2,438 | 0.50 |  |
| Vaccine × Timepoint | 0.56 (0.5 to 0.6) | 271.37 | 2,434.3 | 3e-77 | *** |
| Vaccine × Timepoint × Age | 0 (0 to 0.02) | 1.06 | 2,436.5 | 0.35 |  |
| Vaccine × Timepoint × Baseline S IgG | 0 (0 to 0) | 0.05 | 2,436.1 | 0.95 |  |
| Vaccine × Timepoint × BMI | 0.01 (0 to 0.03) | 1.45 | 2,434.4 | 0.23 |  |
| Vaccine × Timepoint × Sex | 0 (0 to 0) | 0.06 | 2,435 | 0.94 |  |

**Table S3**. **Results from sensitivity analysis excluding anti-spike and anti-nucleocapsid positive individuals.** Individuals who were positive for anti-spike antibodies at baseline (n=20) or anti-nucleocapsid antibodies at 6-month follow-up (n=4), or had missing anti-nucleocapsid antibody data at 6-month follow-up (n=10) were excluded, leaving n=464, and a model was fit with the same structure as the main model. Partial eta squared (η_p_^2^) statistics were calculated F statistics and corresponding degrees of freedom (Df). F statistics were calculated using Type II sums of squares, and thus each compares the model with a specific predictor but without any higher-order predictors to the model without the specific predictor.

| **Predictor** | **Partial η_p_^2^** | **F** | **Df** | **p** | **Signif** |
| --- | --- | --- | --- | --- | --- |
| Age | 0.03 (0.01 to 0.07) | 15.98 | 1,474.7 | 7e-05 | *** |
| Baseline S IgG | 0.12 (0.07 to 0.17) | 72.35 | 1,540.9 | 2e-16 | *** |
| BMI | 0 (0 to 0.01) | 0.06 | 1,488.8 | 0.81 |  |
| Sex | 0.02 (0 to 0.05) | 9.49 | 1,479.6 | 0.002 | ** |
| Smoking status | 0.01 (0 to 0.04) | 7.75 | 1,525.2 | 0.006 | ** |
| Timepoint | 0.61 (0.55 to 0.65) | 665.96 | 1,434.5 | 1e-89 | *** |
| Timepoint × Age | 0 (0 to 0.01) | 0.15 | 1,434.4 | 0.70 |  |
| Timepoint × Baseline S IgG | 0 (0 to 0.02) | 0.76 | 1,488.4 | 0.38 |  |
| Timepoint × BMI | 0.01 (0 to 0.04) | 5.61 | 1,445 | 0.018 | * |
| Timepoint × Sex | 0 (0 to 0.01) | 0.40 | 1,439.2 | 0.53 |  |
| Vaccine | 0.38 (0.31 to 0.43) | 142.75 | 2,475.5 | 3e-49 | *** |
| Vaccine × Age | 0.02 (0 to 0.05) | 4.35 | 2,472.4 | 0.013 | * |
| Vaccine × Baseline S IgG | 0.01 (0 to 0.03) | 2.01 | 2,547.5 | 0.14 |  |
| Vaccine × BMI | 0.01 (0 to 0.04) | 3.77 | 2,518.1 | 0.024 | * |
| Vaccine × Sex | 0.01 (0 to 0.03) | 1.59 | 2,477.6 | 0.20 |  |
| Vaccine × Timepoint | 0.53 (0.47 to 0.58) | 239.93 | 2,433.2 | 7e-71 | *** |
| Vaccine × Timepoint × Age | 0 (0 to 0.01) | 0.11 | 2,433.6 | 0.89 |  |
| Vaccine × Timepoint × Baseline S IgG | 0.03 (0 to 0.06) | 6.77 | 2,493.5 | 0.001 | ** |
| Vaccine × Timepoint × BMI | 0 (0 to 0.02) | 0.62 | 2,475.5 | 0.54 |  |
| Vaccine × Timepoint × Sex | 0 (0 to 0.01) | 0.20 | 2,437.3 | 0.82 |  |

**Table S4**. **Results from sensitivity analysis excluding additional observations possibly affected by boosters.** Observations for the 6-month time point were excluded from analysis for individuals whose latest information regarding which vaccines they received was transmitted to investigators before the 6-month follow-up, and who may therefore have received a booster before the 6-month follow up. Partial eta squared (η_p_^2^) statistics were calculated F statistics and corresponding degrees of freedom (Df). F statistics were calculated using Type II sums of squares, and thus each compares the model with a specific predictor but without any higher-order predictors to the model


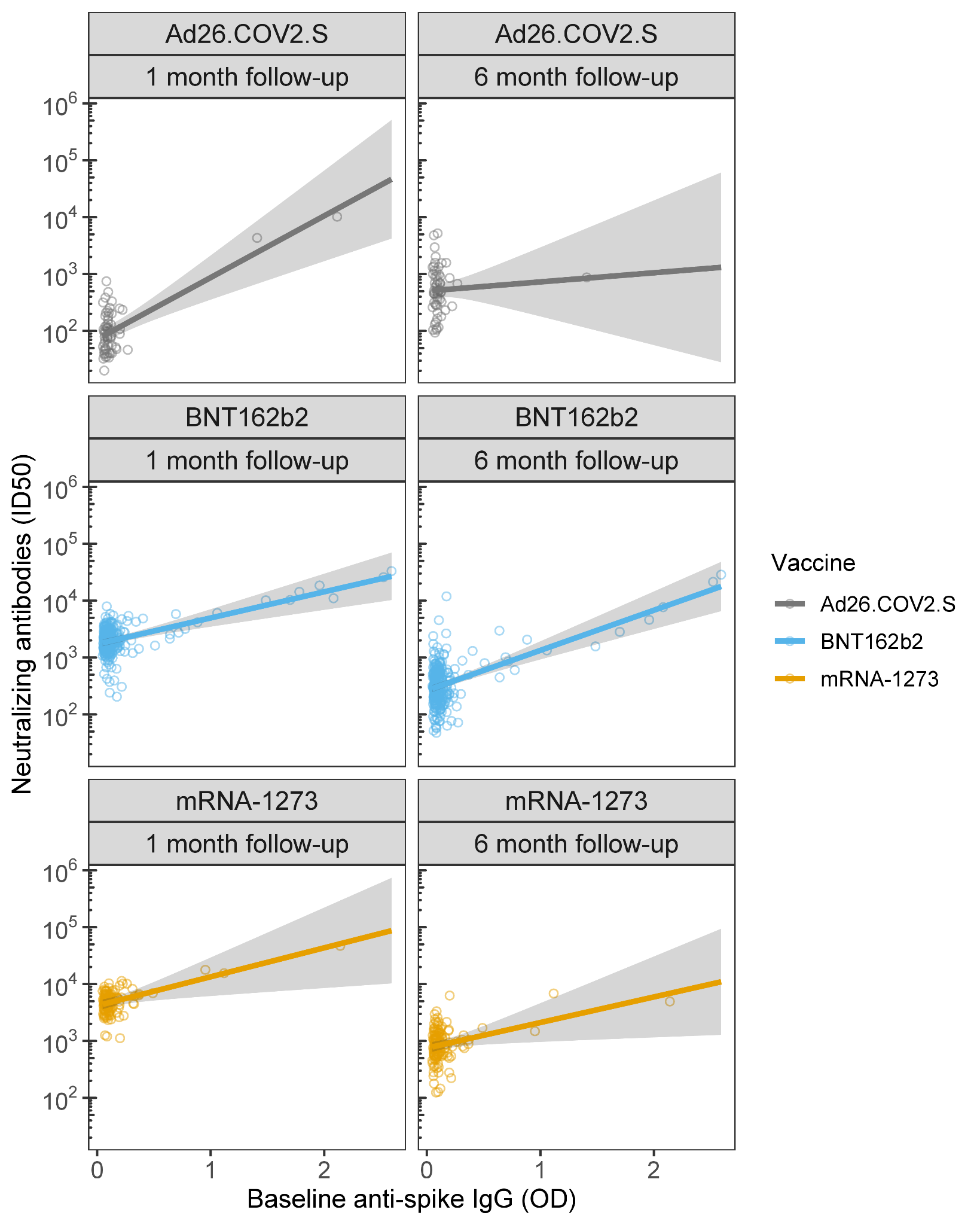


**Figure S1. Time, vaccine, and baseline anti-spike IgG interacted to predict neutralizing antibody level (nAB).** In order to adjust our other tests and estimates for the impact of pre-vaccination exposure to SARS-CoV-2, we included a three-way interaction between time, vaccine, and baseline anti-spike IgG optical density (OD) in our model, which was significant. Lines and shading represent model-derived estimated means +/- 95% CI for all observed levels of baseline spike IgG.
